# Sensitivity Patterns of Bacterial Pathogens Isolated from Blood Cultures of Under-Five Children with Pneumonia and Clinical Sepsis

**DOI:** 10.3390/life11050450

**Published:** 2021-05-18

**Authors:** Sufia Islam, Ashiqur Rahman Akand, Tasnova Tasnim Nova, Christian Lehmann, Mohammod Jobayer Chisti

**Affiliations:** 1 Department of Pharmacy, East West University, Dhaka 1212, Bangladesh; shuvo.akand@gmail.com (A.R.A.); ttm@ewubd.edu (T.T.N.); 2 Department of Anesthesia, Pain Management and Perioperative Medicine, Faculty of Medicine, Dalhousie University, Halifax, NS B3H 1X5, Canada; chlehmann@dal.ca; 3 International Centre for Diarrhoeal Disease Research, Bangladesh, Nutrition & Clinical Services Division, Dhaka 1212, Bangladesh; chisti@icddrb.org

**Keywords:** bacterial resistance, children, mortality, pneumonia, sepsis

## Abstract

Treatment options for pneumonia and sepsis by antibiotics are limited due to the development of multidrug-resistant bacterial strains. This unmatched case-control study determined the antibiotic sensitivity against bacterial isolates obtained from septic and nonseptic children with pneumonia. Children of either sex aged 0–59 months with a history of cough or shortness of breath and radiologically confirmed pneumonia were enrolled in this study. Cases with clinical signs of sepsis at admission (*n* = 151) were compared to cases without sepsis as controls (*n* = 107). A total of 205 children had a performance of blood culture, with 123 children suffering from clinical sepsis. Blood cultures showed bacterial growth in 19% of the septic samples, with 8% coagulase-negative *staphylococci* and 2.4% *Acinetobacter* species. Only 1.6% of the cases were infected by *Streptococcus pneumonia*, *Haemophilus influenzae*, *Salmonella typhi* and *Klebsiella*. In contrast, children without sepsis presented positive blood cultures with growth of *Salmonella typhi* in 2.4% of the cases and growth of *Klebsiella* in 1.2%. Bacteria were sensitive to imipenem in 100% of the cases (86% for meropenem, 83% for ceftazidime and 76% for ciprofloxacin). The mortality rate was significantly higher in children with pneumonia complicated by sepsis (odds ratio (OR) = 3.02, 95% confidence interval (CI), 1.11–8.64, *p* < 0.027). Knowledge about specific laboratory characteristics in children with pneumonia will facilitate an early diagnosis and treatment of sepsis and reduce mortality.

## 1. Introduction

Pneumonia represents a major infectious disease in developing countries and is associated with high morbidity and mortality in children under five years of age [1]. The most important causative bacterial pathogens for childhood pneumonia are *Streptococcus pneumoniae* and *Haemophilus influenzae* type B (Hib) [2]. Among the 5.3 million deaths in children under five years of age worldwide every year, 15% are due to pneumonia [1]. Half of the fatal cases were related to sepsis [1]. Previous studies have reported a 12–26.4% mortality in children with pneumonia in hospitals in Dhaka and Mirzapur, respectively [3,4]. Sepsis is the most common cause of death in infants and children [5]. Data from three slum areas of Dhaka city also showed 20% neonatal deaths due to sepsis [6]. Chisti et al. reported that the case fatality rate was significantly increased in children under five years of age with severe acute malnutrition and pneumonia along with severe sepsis when compared to children without sepsis (40% vs. 4%; *p* < 0.001) [7]. Both pneumonia and sepsis in children still remain the highest burden in developing countries. 

The guidelines of the Surviving Sepsis Campaign recommend antibiotic therapies and fluid resuscitation, which may reduce the mortality and morbidity from sepsis [8,9,10]. However, those guidelines are often not feasible in middle- and low-income countries. The main reason is the lack of resources for treating these sepsis patients [7]. Pneumonia along with sepsis is a medical and financial burden in developing countries. Therefore, early diagnosis followed by prompt therapeutic intervention is needed in order to treat children successfully. Simple clinical and laboratory characteristics can be helpful for early diagnosis in poor or inadequate hospital settings and has to be followed by adequate therapeutic interventions. However, antibiotic resistance is increasing [11]. The options for antibiotic treatment are limited due to the development of resistant *S. pneumoniae* and other multidrug-resistant (MDR) bacterial strains [12,13]. There are reports of *S. pneumoniae* resistant to ampicillin (12%), azithromycin (51.0 %) and erythromycin (51.0%) [12]. β-lactam-, macrolide-, fluoroquinolone- and telithromycin-resistant *S. pneumoniae* were identified in different studies [12,14,15]. Carbapenemase-producing *Klebsiella pneumoniae* are found to be resistant to tigecycline, colistin and aminoglycosides as well [16]. Multidrug-resistant extended-spectrum β-lactamase (ESBL) *Klebsiella pneumoniae* were reported in ICU patients in China [17]. Highly resistant *S. pneumoniae* were also found to be resistant to macrolides [18]. 

Pneumonia and sepsis contribute to a considerable number of child deaths in developing countries including Bangladesh. Therefore, it is very important for clinicians and other healthcare workers to know about the clinical characteristics of pneumonia and sepsis. The determination of simple clinical characteristics has the upmost importance in the case of a poor hospital setting or where the adequate diagnostic resources are not available. This will facilitate early treatment and reduce morbidity and mortality of children. 

Empirical antibiotic therapy is usually prescribed if signs of systemic illness occur. However, it is also important to identify the bacterial pathogens responsible for the clinical condition of the patients. Hence, the sensitivity pattern to antibiotics should be determined for an appropriate treatment of septic patients.

Early diagnosis followed by a prompt therapeutic intervention is needed in order to save children from the complications of pneumonia. Therefore, this study was conducted to describe the antibiotic sensitivity pattern and general laboratory characteristics of septic children under five years of age suffering from pneumonia. 

## 2. Patients and Methods

### 2.1. Design

This was an unmatched case-control study from the patients’ record (September to December 2007) from the Dhaka Hospital of the International Centre for Diarrhoeal Disease Research, Bangladesh (icddr,b)).

### 2.2. Participants

Children of either sex, aged 0–59 months, admitted to the Dhaka Hospital of icddr,b with a history of cough or difficult breathing and radiologically confirmed pneumonia were enrolled in the study. 

### 2.3. Study Procedures

Children were assessed for sepsis and assigned accordingly to a sepsis or control group. 151 patients with clinical sepsis were considered as cases and 107 patients without clinical sepsis were considered as controls. Sepsis was defined if a child had a presumed infection plus tachycardia plus hypothermia (≤35.0 °C) or hyperthermia (≥38.5 °C), plus a capillary refilling time ≥3 s or an abnormal white blood cell (WBC) count in the absence of clinical dehydration [19]. An abnormal WBC count was defined if the WBC count was >11,000/cc, or <4000/cc, or a band and neutrophil ratio ≥ 0.1, or a band >10%. Relevant data about the clinical characteristics collected from the patients included age, sex, vitamin A administration within the last six months, Bacille Calmette Guerin (BCG) vaccination status, clinical dehydration, hypoxemia (SpO_2_ < 90%), hypothermia (body temperature ≤ 35 °C), systemic inflammatory response syndrome (SIRS), history of measles and antibiotic use before admission. The nutritional status (height for age Z scores, weight for age Z scores and weight for height Z scores) of each patient was determined. The laboratory characteristics collected from the patients included WBC, differential count, serum creatinine, sodium, potassium, glucose and bacterial growth in blood. Antibiotic sensitivity, resistance and intermediate sensitivity of bacteria isolated from the blood culture were determined. 

### 2.4. Blood Cultures and Antimicrobial Sensitivity Testing

Two mL of fresh venous blood were collected from the patients on the day of admission with all aseptic precautions and were seeded directly into BacT/ALERT culture bottles. They were loaded into the BacTAlert 3D system. Only one blood sample for each of the participants was collected and tested because of resource constraints.

Antibiotic sensitivity testing was carried out using disk diffusion as recommended by the Clinical Laboratory Standards Institute (CLSI). During the study period, the laboratory followed the available updated editions of the CLSI guidelines (CLSI-2014, CLSI-2015, CLSI-2016, CLSI-2017) [20]. Commercial antimicrobial discs (Oxoid, Basingstoke, United Kingdom) were used for the antibiotic sensitivity test. The zone of inhibition was measured by using the CLSI guidelines, and the antibiotics were marked accordingly as “sensitive“, “intermediate“ or “resistance“ (SIR). Minimum inhibitory concentrations (MICs) were not performed due to limited resources. All reports of culture and sensitivity were available by 48–72 h of sample collection.

### 2.5. Data Analysis

All data were entered into SPSS for Windows (version 15.0; SPSS Inc., Chicago, IL, USA). Differences in proportion were compared by using a Chi-square test. A Student’s *t*-test was done to compare the means of normally distributed data. A Mann–Whitney test was performed to compare the means of data that were not normally distributed. The strength of association was determined by calculating odds ratio (OR) and 95% confidence interval (CI). *p* < 0.05 was considered statistically significant. 

### 2.6. Ethical Considerations

The approval of this chart analysis was waived for the publication by the Ethical Review Committee of icddr,b without any formal registration.

## 3. Results

### 3.1. Blood Cultures

Various bacterial isolates were obtained from the blood of children under five years of age with sepsis and without sepsis. A total of 205 children had a performance of blood culture. Among the 123 children suffering from clinical sepsis, 8% presented coagulase-negative *Staphylococci*, whereas in the nonseptic control group the number was 3.6%. The second most frequently found microorganism in the sepsis group was *Acinetobacter* (2.4%). *Acinetobacter* spp. were not found in the control group. Only 1.6% of cases were infected by *Streptococcus pneumoniae*, *Haemophilus influenzae, Salmonella typhi* and *Klebsiella* spp. in the sepsis group. In contrast, children without sepsis presented a 2.4% growth of *Salmonella typhi*, 1.2% of *Klebsiella* spp. and no growth of *Streptococcus pneumoniae* and *Haemophilus influenzae*. Species of *E. coli* (0.8%) and *Enterococcus* (0.8%) were also obtained in the cultures (Table 1).

### 3.2. Antibiotic Sensitivity

Twenty-nine bacterial cultures of each group were tested for sensitivity to seven antibiotics. Among them, 100% sensitivity was obtained for imipenem, 86% for meropenem, 83% for ceftazidime, 76% for ciprofloxacin, 69% for netilmicin, 72% for amikacin and 52% for gentamicin (Table 2). 

### 3.3. Clinical and Laboratory Characteristics

Sepsis cases were more often found in younger children of <2 months of age. Children under five years old suffered more from clinical dehydration (*p* = 0.018) and hypoxemia (*p* = 0.001). Children without BCG vaccination were more prone to developing sepsis. The edematous malnutrition and weight-to-height ratio were not significantly different between sepsis cases and controls. Significant differences were observed in the total WBC count (*p* = 0.004) and the number of immature poly (*p* = 0.044) between sepsis cases and controls. Blood glucose levels were less divergent between sepsis cases and controls (Table 3). The growth in blood cultures was significantly different in sepsis cases compared to control (*p* = 0.037). The fatality rate was significantly higher among sepsis cases when compared to controls (*p* = 0.027) (Table 3).

## 4. Discussion

Our study evaluated the etiology of pneumonia and the sensitivity pattern of bacterial pathogens isolated from blood cultures of children under five years of age with and without clinical sepsis. A significantly higher case fatality was observed in children with sepsis when compared to those without sepsis.

The organisms most commonly involved in pneumonia are *Streptococcus pneumoniae*, *Haemophilus influenzae* and *Klebsiella pneumoniae*. Downie et al. described *Staphylococcus aureus*, *Escherichia coli* and *Klebsiella* species as the most common pathogens in community-acquired neonatal and infant bacterial infections in developing countries [21]. The most important causative bacterial pathogens for childhood pneumonia are *Streptococcus pneumoniae* and *Haemophilus influenzae* type B (Hib) [2]. The commonly isolated bacterial pathogens found in our study included *Coagulase-negative Staphylococcus* (CoNS), *Klebsiella pneumoniae*, *Streptococcus pneumoniae*, *Acinetobacter* species and *Haemophilus influenzae*. 

The development of multidrug resistance in patients increases the risk of death, and this can be due to the inappropriate use of antibiotics [11,13,22]. In our study, we have found a high level of resistance against gentamicin (48%), ciprofloxacin (17%) and ceftazidime (10%). Imipenem was 100% sensitive in the bacterial cultures of our patients with pneumonia and sepsis. The case fatality of the pneumonia children with sepsis was 15%. 

Although imipenem (100%) and ceftazidime (83%) show sensitivity against bacteria, the costs of these drugs are still very high. When sensitive antibiotics become unaffordable, the mortality rate in patients from low- and middle-income countries (LMIC) will increase. The resistance to other antibiotics, such as 48% resistance to gentamicin and 28% and 21% resistance to netilmicin and amikacin is hazardous for children at this early age. High rates of bacterial resistance have also been observed in other studies on sepsis in neonates and infants in developing countries [21]. Low percentages of intermediate sensitivities of the antibiotics were observed in this study. 

In another study, a total of 404 severely acute malnourished (SAM) children admitted to the Dhaka Hospital of icddr,b were investigated. Twelve percent had acute watery diarrhea, hypernatremia, hypoxemia as well as hypocalcemia [7]. In our study, the clinical and laboratory characteristics of children with pneumonia and sepsis included hypoxemia, hypothermia, hypoglycemia and edematous malnutrition. In the abovementioned study, the case fatality rate in children with severe sepsis and septic shock was between 40% and 69% [7]. The case fatality was 15% in our study. Patients suffering from pneumonia, diarrhoea and dehydration were more often admitted with severe malnutrition and cyanosis. They often showed severe drowsiness [23]. Our study’s children had dehydration and edematous malnutrition. However, we have not observed cyanosis in these children. 

The useful effects of BCG vaccines in patients with nontubercular illness have already been reported by other investigators [24,25]. We have also found an association between the lack of BCG vaccination and sepsis in our study population.

Anubha et al. reported that there was an increasing trend of bacteremia caused by *Staphylococcus aureus* in hospitalized children (one month to 59 months) suffering from pneumonia or sepsis. Our study corroborates the findings of Anubha et al. that CoNS is the main differentiating organism compared to other isolated pathogens [26].

One of the limitations of our study was the lack of performance of a second blood culture, and thus the rate of CoNS infections was most likely overestimated since contamination could not be ruled out. In contrast, the rate of *S. pneumoniae* bacteremia was most likely underestimated. Another limitation of the study is that sputum cultures were not performed due to the unavailability of adequate resources. However, collecting sputum from very sick children can be challenging.

In conclusion, bacteria were isolated in blood cultures from pneumonia patients with and without sepsis with an increased mortality in the sepsis group. CoNS were the main pathogens found in our study. This is an important observation and requires further studies. The significance of the study is the observation of multidrug-resistant gram negatives and CoNS, which may have a future impact for policy makers in revising the national antibiotic guideline for the management of such children, especially in resource-poor settings.

## Figures and Tables

**Table 1:** Isolated bacteria in blood cultures from children under five years of age presenting with and without clinical sepsis.

Organism	Clinical Sepsis (*n* = 123)	without Clinical Sepsis (*n* = 82)
*Streptococcus pneumoniae*	2 (1.6)	0
CoNS ^a^	10 (8.0)	3 (3.6)
*Haemophilus influenzae*	2 (1.6)	0
*Salmonella typhi*	2 (1.6)	2 (2.4)
*Klebsiella species*	2 (1.6)	1 (1.2)
*Acinetobacter* species	3 (2.4)	0
*Escherichia coli*	1 (0.8)	0
*Enterococcus* species	1 (0.8)	0

Values represent *n* (%), ^a^ CoNS = coagulase-negative *staphylococci.*

**Table 2:** Antibiotic sensitivity, resistance and intermediate sensitivity of bacteria isolated from blood cultures in children under five years of age (*n* = 29).

Drugs	Sensitivity (%)	Resistance (%)	Intermediate Sensitivity (%)
Gentamicin	15 (52)	14 (48)	0 (0)
Ciprofloxacin	22 ( 76 )	5 ( 17 )	2 (7)
Ceftazidime	24 ( 83 )	3 (10)	2 (7)
Imipenem	29 (100)	0 (0)	0 (0)
Netilmicin	20 (69 )	8 ( 28 )	1 (3)
Amikacin	21 ( 72 )	6 (21 )	2 (7)
Meropenem	25 (86)	2 (7)	2 (7)

**Table 3:** Clinical and laboratory characteristics of children under five years of age with sepsis (cases) and without sepsis (controls).

Variables	Cases (*n* = 151)	Controls (*n* = 107)	OR	95% CI	*p* Value
Male gender	88 (58)	59 (55)	1.14	0.67–1.93	0.708
Age in months (Median, IQR) **	5.0(0.1,59.0)	8.5 (0.25,59.0)	-	-	0.001 *
No use of antibiotic before admission	78/143/(55)	45 (42)	1.65	0.97–2.83	0.068
<2 months of age	41 (27)	14 (13)	2.48	1.22–5.10	0.010 *
Clinical dehydration(some /severe)	83 (55)	42 (39)	1.89	1.11–3.23	0.018 *
Lack of BCG vaccination	15 (10)	36 (34)	3.79	1.78–8.0	0.001 *
Hypoxemia (SpO2 < 90%)	71 (47)	48 (45)	3.09	2.08–6.85	0.001 *
Hypothermia on or after admission (Temp ≤ 35 °C)	7 (5)	1 (1)	5.15	0.62–113.10	0.145
EdematousMalnutrition	15 (10)	11 (10)	0.96	0.40–2.36	0.905
WHZ (<−3 z score)	41 (27)	20 (19)	1.57	0.82–3.00	0.191
Total WBC count (number/mm^3^; median, IQR) **	15,000 (10,000, 21,100)	12,000 (9000, 16,000)	-	-	0.004 *
Immature poly (number/mm^3^; median, IQR) **	00(00,1.00)	00(00,00)	-	-	0.044 *
Hypoglycemia (RBS < 3 mmo1/L)	20(13)	7(7)	2.21	0.84–6.03	0.118
Bacterial growth in blood culture	23/123(19)	6/82(7)	2.91	1.06–8.44	0.037 *
Outcome (Died)	23(15)	6(6)	3.02	1.11–8.64	0.027 *

Values represent *n* (%) unless specified. OR: odds ratio. CI: confidence interval. IQR: interquartile range. Weight for height z score; SpO2 = transcutaneously measured blood oxygen concentration. * *p* < 0.05 is significant. ** Comparison of age, total WBC count and immature poly (median, IQR) between sepsis cases and controls was done by a Mann–Whitney test that only determined the *p* value; there is no information about the OR and their CIs. RBS: Random blood sugar.

## Data Availability

The data are available with the Research Administration (RA), icddr,b (www.icddrb.org accessed on 18 May 2021) and will be available on request to Armana Ahmed (aahmed@icddrb.org), Head, RA, icddr,b.
